# Association between regular dental scaling and stroke risk in patients with periodontal diseases: evidence from a Korean nationwide database

**DOI:** 10.4178/epih.e2025020

**Published:** 2025-04-19

**Authors:** Yu-Rin Kim, Minkook Son, Seon-Rye Kim

**Affiliations:** 1 Department of Dental Hygiene, Silla University, Busan, Korea; 2 Department of Physiology, Dong-A University College of Medicine, Busan, Korea; 3 Institute of Health Medical Education Convergence Research, Kangwon National University, Samcheok, Korea

**Keywords:** Stroke, Periodicity, Dental scaling, Periodontal diseases

## Abstract

**OBJECTIVES:**

This study aimed to evaluate the association between the frequency of dental scaling and the risk of stroke among individuals with moderate-to-severe periodontal diseases and verify the effect of regular dental scaling on stroke risk in this population.

**METHODS:**

In this retrospective study, 25,758 subjects with moderate-to-severe periodontal diseases were selected from the Korean National Health Insurance Service-National Health Screening Cohort database. Based on the frequency of dental scaling, the subjects were divided into three groups: regular, occasional, and infrequent. Restricted cubic splines were used to evaluate hazard ratios (HRs) with 95% confidence intervals (CIs) for stroke. Additionally, landmark analysis was conducted to strengthen the reliability of the results.

**RESULTS:**

There were 293, 111, and 38 stroke cases in the infrequent, occasional, and regular group, respectively. The adjusted HR for stroke in the regular group, compared to that in the infrequent group, was 0.40 (95% CI, 0.29 to 0.57). In the landmark analysis with follow-up after 1 year and after 2 years, the adjusted HR in the regular group compared to that in the infrequent group was 0.41 (95% CI, 0.28 to 0.60) and 0.50 (95% CI, 0.33 to 0.76), respectively.

**CONCLUSIONS:**

Regular dental scaling was significantly associated with a reduced risk of stroke in patients with moderate-to-severe periodontal diseases. These findings may suggest a potential preventive role of dental scaling beyond oral health. Further studies are needed to explore the underlying biological mechanisms linking periodontal care to stroke prevention and to explore causal relationships between dental scaling and stroke risk.

## GRAPHICAL ABSTRACT

[Fig f5-epih-47-e2025020]

## Key Message

Among adults with moderate to severe periodontitis, those who underwent regular dental scaling had a significantly lower risk of stroke compared to those without routine scaling. This protective effect was especially evident in males and individuals aged 65 and older. These results suggest that regular oral healthcare may serve as a potential contributing factor in the prevention of cerebrovascular diseases.

## INTRODUCTION

Stroke is an ischemic condition characterized by a sudden loss of blood flow to the brain, resulting in loss of neurological function. The primary cause of stroke is an embolism, including thrombosis [1]. Stroke is the second leading cause of death and the third leading cause of disability worldwide [2]. Notably, most risk factors for stroke are modifiable [3], and proper interventions targeting these modifiable risk factors may significantly reduce the risk of stroke.

Oral health plays a vital role in overall health. Periodontal diseases, one of the most common inflammatory diseases globally, is caused by various bacteria and adversely affects the supportive structures of teeth [4,5]. It can lead to transient bacteremia, systemic inflammation, and endothelial dysfunction [6]. Chronic inflammation plays a crucial role in the pathogenesis of both periodontal diseases and stroke, as it contributes to atherosclerosis, arterial stiffness, and thrombus formation, which are key mechanisms underlying ischemic stroke [7]. Additionally, specific oral pathogens involved in periodontal diseases, such as *Porphyromonas gingivalis*, have been detected in atherosclerotic plaques, further supporting the link between periodontal diseases and vascular diseases [8].

The systemic inflammatory burden caused by periodontal diseases may contribute to cerebrovascular pathology through multiple pathways, including increased levels of pro-inflammatory cytokines (e.g., interleukin-6 [IL-6] and, tumor necrosis factor-alpha [TNF-α]), oxidative stress, and endothelial dysfunction [9]. Furthermore, periodontal diseases-related bacteremia may promote platelet aggregation and clot formation, thereby increasing the risk of thromboembolic events [10]. Given these mechanisms, maintaining good periodontal health may serve as an important preventive measure against cerebrovascular diseases.

Dental scaling is a representative preventive dental treatment for periodontal diseases such as periodontal diseases [11], and regular professional cleaning has been shown to reduce periodontal diseases [12,13]. Several case–control and prospective cohort studies have explored the association between periodontal diseases and stroke [14-17]. Systemic inflammatory reactions resulting from the invasion of oral bacteria or inflammatory mediators produced in response to periodontal disease or poor oral hygiene may increase the risk of stroke [18]. Therefore, elucidating this possible link is of paramount importance. Furthermore, Lee et al. [19] reported that managing oral health through periodontal treatment could reduce the incidence of ischemic stroke. However, only a few studies have investigated the causal relationship between periodontal treatment and stroke [12,19]. Therefore, whether dental treatment has protective effects against cerebrovascular diseases remains unclear. Additionally, no studies have examined whether regular dental scaling affects the incidence of stroke among patients with periodontal diseases.

Previous studies have several limitations, including heterogeneous definitions of periodontal disease, lack of consideration of potential confounders such as socioeconomic status, small sample sizes, and insufficient statistical power. Moreover, some studies have overestimated the prevalence of periodontal disease because of their reliance on self-reported data or non-standardized diagnostic criteria [14-17].

To address these gaps, this study used a large-scale, nationally representative cohort database, the National Health Insurance Service-National Health Screening Cohort (NHIS-HEALS), which enabled a more comprehensive assessment of the relationship between regular dental scaling and stroke incidence. Unlike previous studies, our study applied standardized diagnostic and treatment codes for periodontal diseases, ensuring a more precise and clinically relevant definition of disease status. Furthermore, the NHIS-HEALS database provided extensive demographic and clinical information, allowing us to adjust for a wide range of potential confounders, including age, sex, income level, residence, hypertension, diabetes, dyslipidemia, myocardial infarction, depression, and Charlson comorbidity index (CCI).

By leveraging this robust dataset, our study offers significant methodological advantages over previous studies, addressing the limitations related to sample size, confounding variables, and disease classification. In particular, we investigated the impact of regular dental scaling on stroke incidence among individuals with periodontal diseases by analyzing the frequency of dental scaling during the follow-up period. The use of a nationwide database strengthens the generalizability of our findings and, provides valuable insights into the potential benefits of preventive dental care in reducing the risk of stroke in this population.

## MATERIALS AND METHODS

### Study population

This study utilized data from the Korea NHIS-HEALS cohort database, which includes individuals aged≥40 years who participated in the NHIS health screening programs. The NHIS-HEALS database contains diagnoses based on the International Classification of Diseases, 10th revision (ICD-10), medical procedures, and prescribed medications. Subjects were monitored from January 1, 2002, to December 31, 2019.

Patients with moderate-to-severe periodontal disease (n=25,758) were identified in July 2013 following the implementation of the dental scaling reimbursement policy, with the preceding period designated as the washout period. Post-policy, participants were categorized into three groups based on the frequency of dental scaling: regular (frequency>0.8), occasional (frequency between 0.3 and 0.8); and, infrequent (frequency<0.3). The final analysis included 5,521 participants in the regular group; 9,495, occasional; and, 10,742, infrequent. To enhance the reliability of stroke risk assessment, landmark analyses were conducted beginning one year (regular group=5,514; occasional group=9,476; infrequent group=10,575) and two years (regular group=5,510; occasional group=9,447; infrequent group=10,343) was implemented.

**Figure 1.**
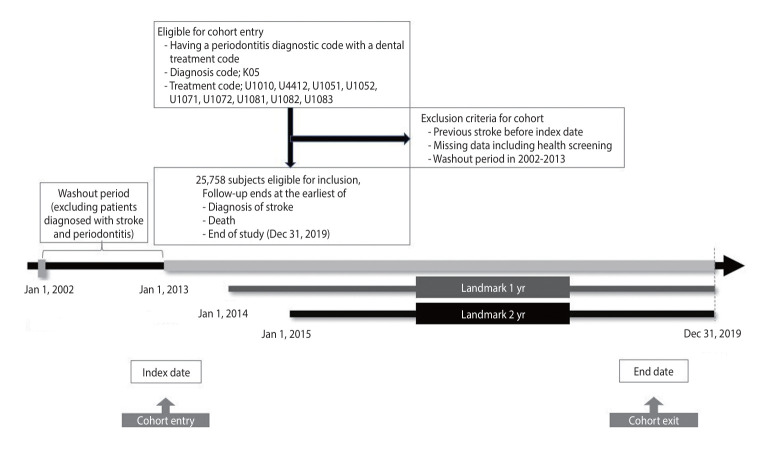
The flow of study population.

We set the washout period from January 1, 2002, to June 30, 2013, to ensure that only individuals who received dental scaling after it became covered by the national health insurance system were included in our analysis. The rationale for selecting July 2013 as the starting point was that dental scaling procedures began to be systematically recorded in the national health insurance claims database only after insurance coverage was introduced. This approach strengthened the validity of our study by reducing potential biases associated with undocumented or inconsistent recording of scaling procedures before this period. By ensuring that our study population consisted only of individuals whose scaling history was reliably captured, we enhanced the robustness of our findings regarding the association between dental scaling and stroke risk (Figure 1).

### Definition of moderate-to-severe periodontal diseases

The number of periodontal diseases-related dental procedures performed between July 2013 and July 2015 was identified using the ICD-10 diagnostic codes for periodontal disease (K05). The treatment code for moderate periodontal diseases was subgingival curettage (U1010). Treatment codes for severe periodontal diseases included tooth extraction (U4412), periodontal flap surgery (U1051, U1052), bone grafting for alveolar bone defects (U1071, U1072), and guided tissue regeneration (U1081-U1083) [20].

### Outcome

Stroke was identified using ICD-10 codes “I60-I64”. Patients with stoke were defined as those with at least one hospitalization and brain imaging, such as computed tomography or magnetic resonance imaging. Ischemic stroke was defined as “I63, I64”, whereas hemorrhagic stroke was defined as “I60-I62” [21].

### Covariates

The baseline characteristics of the study population were extracted from the NHIS database based on the date of each participant’s first periodontal diseases-related procedure. These variables including demographic factors (sex, age, income level, and residential area); major non-communicable diseases such as hypertension “I10, I11”, diabetes “E10-E14”, dyslipidemia “E78”, myocardial infarction “I21, I22”, and depression “F32, F33”; and, CCI, were identified using ICD-10 codes.

### Statistical analysis

Variables were reported as means with standard deviations or frequencies with percentages. Differences in covariates among the three groups were analyzed using a one-way analysis of variance and the chi-square test. The stroke incidence rate was calculated per 1,000 person-years (PY) during the follow-up period.

Kaplan–Meier curves were generated for the stroke risk analysis, and a log-rank test was performed. A Cox proportional-hazards model was used to calculate the hazard ratio (HR) and 95% confidence intervals (CIs) for stroke risk. Covariates, including sex, age, income level, residential area, hypertension, diabetes, dyslipidemia, myocardial infarction, depression, and CCI, were adjusted for in the model. Subgroup analyses considered sex and age. To enhance the comparability of stroke risk across the dental scaling frequency categories, restricted cubic splines were applied to examine the HR trends and their 95% CIs according to the scaling frequency. These plots were used to model the association between the frequency of dental scaling and stroke risk, allowing for a more flexible representation of potential non-linear relationships. These plots provided a smoothed estimate of the HR across different frequencies of dental scaling, aiding in the interpretation of dose-response effects.

Landmark analysis is a methodological approach that designates a specific time point during the follow-up period, referred to as the landmark time, and includes only individuals who survived until that time for the subsequent analyses [22]. This technique helps mitigate immortal time bias by ensuring that exposure classification occurs only among individuals who remain at risk at the landmark time, thus preventing misclassification of person–time. Furthermore, landmark analysis reduces collider bias by minimizing the risk of selecting individuals based on survival-related factors [23,24], which may confound the association between dental scaling and stroke incidence. In this study, the landmark points were set at one year and two years. All statistical analyses were conducted using R version 4.3.0 (R Foundation for Statistical Computing, Vienna, Austria), with statistical significance set at p-value<0.05.

### Ethics statement

As this retrospective study used anonymized claims data, the requirement for informed consent was waived. Ethical approval was obtained from the Institutional Review Board of Youngsan University (YSUIRB-202409-HR-164-02).

## RESULTS

### Baseline characteristics by frequency of dental scaling

This study included 25,758 participants. Table 1 presents the baseline characteristics of the subjects who were, divided into three groups based on dental scaling frequency. Among the subjects with moderate or severe chronic periodontal diseases, 5,521 patients were in the regular group (frequency>0.8); 9,495 occasional (frequency between 0.3 and 0.8); and, 10,742 infrequent (frequency<0.3). As the frequency of dental scaling increased, the mean age of the participants decreased. Moreover, the proportion of individuals with higher income levels and urban residency increased with greater scaling frequency. Conversely, the prevalence of underlying conditions, such as hypertension, diabetes, myocardial infarction, and depression, was higher in groups with a lower scaling frequency. The CCI also decreased at higher scaling frequencies. The mean frequency values for the three groups were 0.1±0.1, 0.4±0.1, and 0.9±0.1, respectively (Table 1).

### Restricted cubic spline analysis of the association between dental scaling frequency and stroke

**Figure 2.**
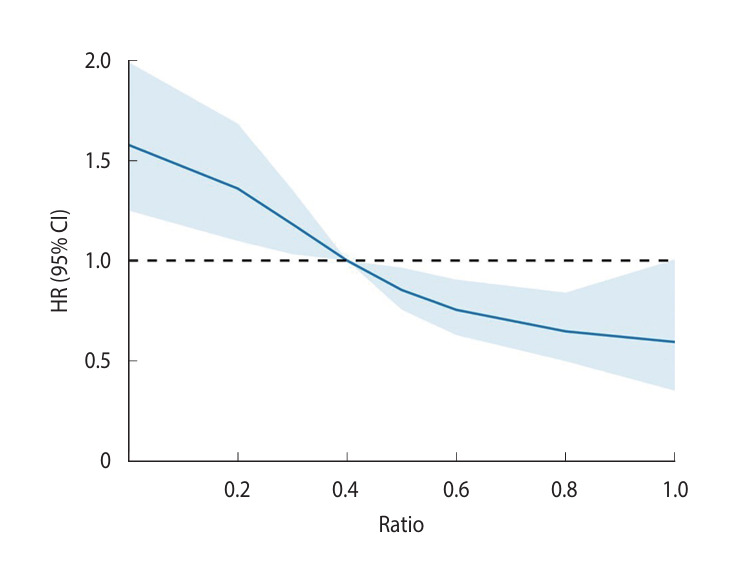
Restricted cubic spline of hazard ratio (HR) with 95% confidence intervals (CIs) for stroke.

As the scaling frequency increased, the HR for stroke decreased. The blue line represents the HR, whereas the shaded area indicates the 95% CIs for stroke incidence. The horizontal dotted line denotes a reference HR of 1.0, and a dental scaling frequency of 0.4 was set as the reference point for the spline plot (Figure 2).

### Stroke risk based on frequency of dental scaling

Among the 25,758 participants, 293, 111, and 38 had stroke events in the infrequent, occasional, and regular groups, respectively. The incidence rate (per 1,000 PY) was the lowest in the regular group (1.39), followed by the occasional (2.39) and infrequent groups (5.58). The crude HRs for stroke were 0.25 (95% CI, 0.18 to 0.35) for the regular group and 0.43 (95% CI, 0.34 to 0.53) for the occasional group, compared to those in the infrequent group. The adjusted HRs were 0.40 (95% CI, 0.29 to 0.57) for the regular group and 0.60 (95% CI, 0.48 to 0.76) for the occasional group.

By stroke type, 269 patients with ischemic stroke were classified into the infrequent group; 105, occasional; and, 36, regular. The incidence rate (per 1,000 PY) of ischemic stroke was lower in the regular group (1.32) compared to that in the infrequent group (5.12). The crude HR for ischemic stroke in the regular group was 0.26 (95% CI, 0.18 to 0.36) compared to that in the infrequent group, with an adjusted HR of 0.43 (95% CI, 0.30 to 0.61). There were 28 cases of hemorrhagic stroke in the infrequent group; 7, occasional; and, 2, regular. The incidence rate (per 1,000 PY) was lower in the regular group (0.07) than that in the infrequent group (0.53). The crude HR for hemorrhagic stroke in the regular group was 0.14 (95% CI, 0.03 to 0.59) compared to that in the infrequent group, with an adjusted HR of 0.19 (95% CI, 0.04 to 0.83) (Table 2).

**Figure 3.**
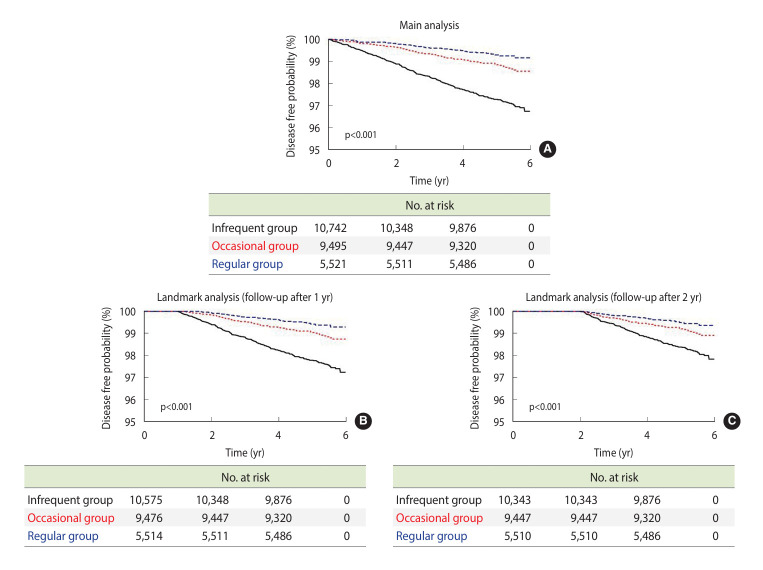
Kaplan–Meier curve for the association between frequency of dental scaling and stroke. (A) Main analysis; (B) Landmark analysis (follow-up after 1 yr); and (C) Landmark analysis (follow-up after 2 yr).

Kaplan–Meier curves were used to visualize the cumulative incidence of stroke between individuals who underwent regular dental scaling and those who did not. These curves illustrate the time-to-event analysis showing the probability of stroke-free survival over the follow-up period. A log-rank test was performed to compare the differences between groups. They demonstrated a significant reduction in the disease-free probability for the regular group compared to that in the infrequent group. The log-rank test yielded a statistically significant p-value of p<0.001. Similarly, the one-year and two-year landmark analyses showed a greater decline in disease-free probability in the regular group, as indicated by Kaplan–Meier curves, both with p-values of p<0.001 (Figure 3).

### Landmark analysis

In the landmark analysis with a follow-up period starting one year post-baseline, among 25,565 participants, 238 stroke cases occurred in the infrequent group; 93, occasional; and, 31, regular group. The incidence was lower in the regular group (1.14) than that in the infrequent group (4.54). The crude HR for stroke in the regular group was 0.25 (95% CI, 0.17 to 0.36) compared to the infrequent group, with an adjusted HR of 0.41 (95% CI, 0.28 to 0.60).

In the two-year landmark analysis, among 25,300 participants, 174 stroke cases occurred in the infrequent group; 77, occasional; and, 27, regular. The incidence rate was lower in the regular group (0.99) than that in the infrequent group (3.34). The crude HR for stroke in the regular group was 0.30 (95% CI, 0.20 to 0.45) compared to that in the infrequent group, with an adjusted HR of 0.50 (95% CI, 0.33 to 0.76; Table 3).

### Subgroup analysis according to sex and age

In the subgroup analysis by age, participants were divided into two groups: those aged ≥65 years and those aged <65 years. Stroke incidence was lower in the regular scaling group than that in the occasional scaling group across all age groups. The adjusted HR for stroke incidence in the regular group was 0.38 (95% CI, 0.23 to 0.62) for individuals aged ≥65 years and 0.46 (95% CI, 0.28 to 0.77) for those aged <65 years.

**Figure 4.**
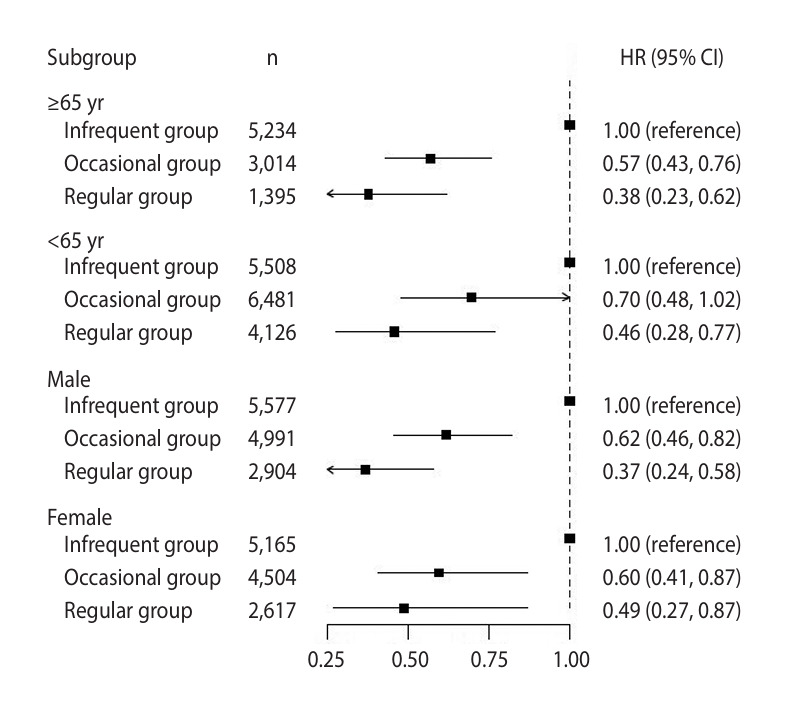
Subgroup analysis by age and sex. HR, hazard ratio; CI, confidence interval.

In the subgroup analysis by sex, the stroke incidence was lower in the regular group than that in the occasional group for both male and female. The adjusted HR for stroke incidence in the occasional group was 0.62 (95% CI, 0.46 to 0.82) for male and 0.60 (95% CI, 0.41 to 0.87) for female. For the regular group, the adjusted HR for stroke was 0.37 (95% CI, 0.24 to 0.58) for male and 0.49 (95% CI, 0.27 to 0.87) for female (Figure 4).

## DISCUSSION

This national retrospective cohort study found that regular dental scaling significantly reduced the incidence of stroke in patients with moderate-to-severe periodontal diseases. This reduction in stroke risk was consistent across both sex and age groups. Additionally, landmark analyses conducted one year and two years after baseline consistently showed that higher frequencies of dental scaling were associated with a lower stroke risk. This association was significant for both the ischemic stroke and hemorrhagic stroke subtypes, reinforcing the robustness of our findings. By applying landmark analysis, we effectively mitigated the immortal time bias, ensuring that the observed association reflected a genuine protective effect rather than an artifact of the study design.

Periodontal disease is a widespread public health issue, with a prevalence of approximately 62% for periodontal diseases and 24% for severe periodontal diseases in adults [25]. Major risk factors for periodontal diseases include income level, education level, smoking, obesity, hyperlipidemia, and diabetes [26], which could be targets for interventions to improve oral health. Periodontitis is associated with a high risk of cardiovascular disease [27]. Moreover, periodontal disease affected the risk of atrial fibrillation [28]. Additionally, periodontal diseases is associated with an elevated risk of stroke [12,15,29-31]. Furthermore, periodontal diseases is a risk factor for ischemic stroke, and periodontal treatments can reduce the stroke risk [19,32]. Consistent with these findings, our study provides further evidence that regular dental scaling is associated with a reduced risk of stroke, including its subtypes, after adjusting for potential confounders such as age, income level, residence, hypertension, diabetes, dyslipidemia, myocardial infarction, depression, and CCI.

The Cox proportional hazards model indicated a lower stroke risk among patients with a higher dental scaling frequency, whereas Kaplan–Meier curves revealed significant differences in disease-free probabilities among the regular, occasional, and infrequent groups, with the regular group exhibiting the highest disease-free probability. These findings suggest that regular dental scaling could be a potential preventive strategy for stroke in patients with periodontal diseases.

Landmark analyses were conducted to minimize bias and confirm the association between dental scaling frequency and stroke incidence. Immortal time bias arises when time-dependent events are improperly analyzed, leading to potentially misleading conclusions about treatment efficacy. Landmark analysis is a key methodological approach that effectively mitigates immortal time bias by ensuring that exposure classification occurs only among individuals who remain at risk at a predefined time point. This prevents the misclassification of person-time and eliminates the artificial survival advantage often introduced in time-to-event analyses. By incorporating landmark analysis, the study provides more reliable estimates of the association between the exposure and outcome. [22-24]. In this study, we implemented landmark analyses one year and two years after baseline. By applying this approach, we assessed the association between dental scaling frequency and stroke risk among individuals with comparable survival characteristics at the designated landmark time points. Both analyses consistently showed a significantly lower risk of stroke in the regular dental scaling group, highlighting the robustness of this association.

Subgroup analyses revealed that regular dental scaling reduced the risk of both ischemic and hemorrhagic stroke compared to that associated with infrequent dental scaling, corroborating previous studies reporting reductions in ischemic stroke risk with dental scaling [12,19,33,34]. Furthermore, analyses by sex and age showed that the preventive effect of regular dental scaling was more pronounced in male and in individuals aged ≥65 years. These findings may be explained by several biological and behavioral factors. Older adults are at a higher risk of both periodontitis and stroke and may experience a greater systemic inflammatory burden due to chronic infections and age-related immune dysregulation [35]. As older adults may already have compromised vascular integrity, reducing the inflammatory load through regular dental scaling may have a more substantial impact on lowering the stroke risk. This supports earlier findings that older adults may derive greater stroke prevention benefits from regular dental care [34]. Similarly, a previous study highlighted the importance of consistent oral hygiene management, such as frequent tooth brushing and professional dental cleaning, in reducing stroke risk [36]. Regarding sex differences, one possible explanation is that male tend to have a higher baseline risk of periodontitis due to poorer oral hygiene behaviors, higher rates of smoking, and greater susceptibility to systemic inflammation compared to those in female [37]. Given that periodontitis-induced inflammation is a key contributor to the risk of stroke, male may experience a greater reduction in systemic inflammation after periodontal care, leading to a more pronounced protective effect. Furthermore, sex-based differences in immune responses may play a role; male exhibit stronger inflammatory responses to chronic infections than female, which may make them more vulnerable to periodontitis-related vascular complications [38]. This could explain why the impact of regular dental scaling on stroke risk reduction was more significant in male. Although our findings suggest that both male and older adults benefit more from regular dental scaling, further research is warranted to explore the precise biological and behavioral mechanisms underlying these differences.

The biological mechanism underlying the association between regular dental scaling and reduced stroke risk likely involves a systemic inflammatory response triggered by periodontal diseases. Periodontal disease induces chronic systemic inflammation, leading to endothelial dysfunction, increased arterial stiffness, and atherosclerosis [27]. The inflammatory mediators produced in periodontal diseases, including IL-6, TNF-α, and C-reactive protein, promote atherogenesis and thromboembolic events, which can contribute to stroke pathogenesis [9]. Additionally, periodontal diseases-related bacteremia may enhance platelet aggregation and vascular inflammation, further increasing the risk of thrombotic stroke [28]. The presence of *P. gingivalis* in atherosclerotic plaques supports the causal link between periodontal pathogens and vascular diseases [26]. By reducing the inflammatory and bacterial loads in the oral cavity, regular dental scaling may help mitigate these systemic effects, thereby lowering the risk of cerebrovascular events.

Globally, stroke remains a major health challenge, with approximately 12 million incident cases and 101 million prevalent cases [2]. Current stroke prevention strategies focus on modifying risk factors, including hypertension, dyslipidemia, diabetes, smoking, and physical inactivity [3,39,40]. Although previous studies have suggested associations between oral hygiene factors (e.g., tooth brushing, dental caries, and tooth loss) and stroke [33,36], few have particularly examined the effects of regular dental scaling on stroke incidence. This study provides robust evidence of a significant negative association between dental scaling frequency and stroke risk after adjusting for potential confounders.

This study has several strengths. This is one of the largest community-based studies to examine periodontal diseases, dental-care utilization, and stroke risk. It uses comprehensive periodontal assessments, validated classifications, and rigorous measurements of confounders. In addition, the analysis considered different severities of periodontal diseases and stroke subtypes. Landmark analysis helped mitigate the immortal time bias and, strengthened the reliability of our findings. However, this study has some limitations. First, certain potential confounders, such as smoking status and genetic factors, were not included in the analysis, despite their known influence on both periodontal diseases and stroke risk. In particular, smoking is a well-established risk factor for both conditions and should be considered in future studies. Second, although we adjusted for socioeconomic factors such as income level and residence, unmeasured confounders, including educational status and blood inflammatory biomarkers, could not be fully accounted for. Additionally, residual confounding cannot be entirely excluded. In particular, we were unable to account for the level of management and control of pre-existing conditions (e.g., blood pressure, blood glucose, and lipid levels), which may substantially influence stroke risk. Since inadequate management of these comorbidities can mediate or modify the association between periodontal health and stroke outcomes. Third, the retrospective nature of the study and reliance on baseline data limited the ability to account for time-dependent factors. Additionally, although landmark analysis was implemented to address immortal time bias and reduce collider bias, it had inherent limitations. Although landmark analysis ensures that individuals are classified into exposure groups only after a defined period, it does not completely eliminate selection bias, as individuals who survive to the landmark time may differ systematically from those who do not. Furthermore, landmark analysis may not fully adjust for residual confounding factors due to unmeasured factors that influence both dental scaling frequency and stroke risk. Finally, because of the observational nature of this study, causal relationships cannot be definitively established. Although our findings suggest a strong association, future randomized controlled trials or Mendelian randomization analyses are required to validate the causal impact of regular dental scaling on stroke risk reduction.

Despite these limitations, this study highlights the importance of regular dental scaling for stroke prevention, particularly in patients with moderate-to-severe periodontal diseases. Regular dental scaling was associated with a significantly lower risk of stroke, irrespective of the stroke subtype, sex, or age. Further studies are needed to explore the underlying biological mechanisms linking periodontal care to stroke prevention and to establish definitive causal relationships that support clinical application.

## Figures and Tables

**Figure f5-epih-47-e2025020:**
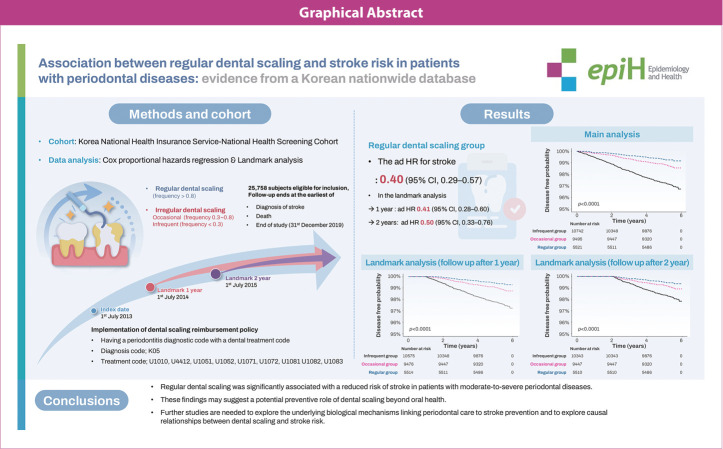


**Table 1.** Baseline characteristics of study population

Characteristics	Infrequent group (n=10,742)	Occasional group (n=9,495)	Regular group (n=5,521)	p-value
Sex				0.580
Male	5,577 (51.9)	4,991 (52.6)	2,904 (52.6)	
Female	5,165 (48.1)	4,504 (47.4)	2,617 (47.4)	
Age (yr)	65.4±9.5	61.8±7.6	60.6±6.8	<0.001
Income level (quartile)				<0.001
1st	1,857 (17.3)	1,468 (15.5)	782 (14.2)	
2nd	2,465 (22.9)	1,998 (21.0)	1,031 (18.7)	
3rd	3,268 (30.4)	2,820 (29.7)	1,484 (26.9)	
4th	3,152 (29.3)	3,209 (33.8)	2,224 (40.3)	
Residence				<0.001
Rural	4,950 (46.1)	3,321 (35.0)	1,436 (26.0)	
Urban	5,792 (53.9)	6,174 (65.0)	4,085 (74.0)	
Hypertension	5,823 (54.2)	4,352 (45.8)	2,399 (43.5)	<0.001
Diabetes	1,909 (17.8)	1,292 (13.6)	651 (11.8)	<0.001
Dyslipidemia	4,042 (37.6)	3,502 (36.9)	2,108 (38.2)	0.260
Myocardial infarction	383 (3.6)	301 (3.2)	151 (2.7)	0.020
Depression	2,730 (25.4)	2,248 (23.7)	1,295 (23.5)	0.003
Charlson comorbidity index				<0.001
0	3,793 (35.3)	3,711 (39.1)	2,174 (39.4)	
1	2,691 (25.1)	2,506 (26.4)	1,532 (27.7)	
2	1,746 (16.3)	1,540 (16.2)	884 (16.0)	
≥3	2,512 (23.4)	1,738 (18.3)	931 (16.9)	
Ratio	0.1±0.1	0.4±0.1	0.9±0.1	<0.001

Values are presented as number (%) or mean±standard deviation.

**Table 2.** Association between frequency of dental scaling and stroke

Group	n	Events (n)	Follow-up duration (PY)	Incidence rate (per 1,000 PY)	Crude HR (95% CI)	p-value	Adjusted^1^ HR (95% CI)	p-value
Composite of outcomes								
Infrequent group	10,742	293	52,507	5.58	1.00 (reference)		1.00 (reference)	
Occasional group	9,495	111	46,517	2.39	0.43 (0.34, 0.53)	<0.001	0.60 (0.48, 0.76)	<0.001
Regular group	5,521	38	27,258	1.39	0.25 (0.18, 0.35)	<0.001	0.40 (0.29, 0.57)	<0.001
Ischemic stroke								
Infrequent group	10,742	269	52,541	5.12	1.00 (reference)		1.00 (reference)	
Occasional group	9,495	105	46,525	2.26	0.44 (0.35, 0.55)	<0.001	0.63 (0.50, 0.79)	<0.001
Regular group	5,521	36	27,262	1.32	0.26 (0.18, 0.36)	<0.001	0.43 (0.30, 0.61)	<0.001
Hemorrhagic stroke								
Infrequent group	10,742	28	53,098	0.53	1.00 (reference)		1.00 (reference)	
Occasional group	9,495	7	46,741	0.15	0.29 (0.13, 0.66)	0.003	0.37 (0.16, 0.87)	0.022
Regular group	5,521	2	27,331	0.07	0.14 (0.03, 0.59)	0.007	0.19 (0.04, 0.83)	0.027

PY, person-years; HR, hazard ratio; CI, confidence interval.

1 The model was adjusted for age, sex, income level, residence, hypertension, diabetes, dyslipidemia, myocardial infarction, depression, and Charlson comorbidity index.

**Table 3.** Association between frequency of dental scaling and stroke

Group	n	Events (n)	Follow-up duration (PY)	Incidence rate (per 1,000 PY)	Crude HR (95% CI)	p-value	Adjusted^1^ HR (95% CI)	p-value
Landmark analysis (follow-up after 1yr)								
Infrequent group	10,575	238	52,411	4.54	1.00 (reference)		1.00 (reference)	
Occasional group	9,476	93	46,506	2.00	0.44 (0.35, 0.56)	<0.001	0.62 (0.49, 0.80)	<0.001
Regular group	5,514	31	27,253	1.14	0.25 (0.17, 0.36)	<0.001	0.41 (0.28, 0.60)	<0.001
Landmark analysis (follow-up after 2 yr)								
Infrequent group	10,343	174	52,060	3.34	1.00 (reference)		1.00 (reference)	
Occasional group	9,447	77	46,460	1.66	0.50 (0.38, 0.66)	<0.001	0.73 (0.55, 0.96)	<0.001
Regular group	5,510	27	27,245	0.99	0.30 (0.20, 0.45)	<0.001	0.50 (0.33, 0.76)	<0.001

PY, person-years; HR, hazard ratio; CI, confidence interval.

1 The model was adjusted for age, sex, income level, residence, hypertension, diabetes, dyslipidemia, myocardial infarction, depression, and Charlson comorbidity index.
